# Treatment of Pediatric Acute Graft-versus-Host Disease—Lessons from Primary Immunodeficiency?

**DOI:** 10.3389/fimmu.2017.00328

**Published:** 2017-03-21

**Authors:** Aisling M. Flinn, Andrew R. Gennery

**Affiliations:** ^1^Medical School, Institute of Cellular Medicine, Newcastle University, Newcastle upon Tyne, UK

**Keywords:** acute graft-versus-host disease, extracorporeal photopheresis, thymopoiesis, dendritic cell, tolerogenesis, primary immunodeficiency

## Abstract

Allogeneic hematopoietic stem cell transplant (HSCT) is used to treat increasing numbers of malignant and non-malignant disorders. Despite significant advances in improved human leukocyte antigens-typing techniques, less toxic conditioning regimens and better supportive care, resulting in improved clinical outcomes, acute graft-versus-host disease (aGvHD) continues to be a major obstacle and, although it principally involves the skin, gastrointestinal tract, and liver, the thymus is also a primary target. An important aim following HSCT is to achieve complete and durable immunoreconstitution with a diverse T-cell receptor (TCR) repertoire to recognize a broad range of pathogens providing adequate long-term adaptive T-lymphocyte immunity, essential to reduce the risk of infection, disease relapse, and secondary malignancies. Reconstitution of adaptive T-lymphocyte immunity is a lengthy and complex process which requires a functioning and structurally intact thymus responsible for the production of new naïve T-lymphocytes with a broad TCR repertoire. Damage to the thymic microenvironment, secondary to aGvHD and the effect of corticosteroid treatment, disturbs normal signaling required for thymocyte development, resulting in impaired T-lymphopoiesis and reduced thymic export. Primary immunodeficiencies, in which failure of central or peripheral tolerance is a major feature, because of intrinsic defects in hematopoietic stem cells leading to abnormal T-lymphocyte development, or defects in thymic stroma, can give insights into critical processes important for recovery from aGvHD. Extracorporeal photopheresis is a potential alternative therapy for aGvHD, which acts in an immunomodulatory fashion, through the generation of regulatory T-lymphocytes (Tregs), alteration of cytokine patterns and modulation of dendritic cells. Promoting normal central and peripheral immune tolerance, with selective downregulation of immune stimulation, could reduce aGvHD, and enable a reduction in other immunosuppression, facilitating thymic recovery, restoration of normal T-lymphocyte ontogeny, and complete immunoreconstitution with improved clinical outcome as the ability to fight infections improves and risk of secondary malignancy or relapse diminishes.

## Introduction

Allogeneic hematopoietic stem cell transplant (HSCT) is used to treat a wide variety of malignant and non-malignant disorders. Significant improvements in human leukocyte antigens (HLA)-typing techniques, less toxic conditioning regimens and better supportive care, have resulted in improved clinical outcomes, with over 90% survival and cure for some diseases. An important aim following HSCT is to achieve durable immune reconstitution (IR) with a diverse T-cell receptor (TCR) repertoire capable of recognizing a broad range of pathogens providing adequate adaptive T-lymphocyte immunity long term. This is essential to reduce the risk of infection, disease relapse, and secondary malignancies (1). Although rebuilding of the innate immune system occurs relatively quickly, reconstitution of adaptive immunity is more complex (2). Complete and long-lasting IR depends on a functioning and structurally intact thymus responsible for the production of naïve T-lymphocytes with a broad TCR repertoire (3). Acute graft-versus-host disease (aGvHD) remains a major obstacle to allogeneic HSCT and, although it principally involves the skin, gastrointestinal tract, and liver, the thymus is also a primary target. The consequent damage to the thymic microenvironment disturbs normal signaling required for thymocyte development and results in impaired thymopoiesis and reduced thymic export. First-line treatment for aGvHD is corticosteroids which have a range of direct effects on many aspects of immunity including thymic function, often followed by a plethora of other agents that non-selectively target T-lymphocytes, further interfering with normal T-lymphocyte neogenesis and subjecting patients to an increased risk of infection and relapse. Targeted therapy for aGvHD without systemic immunosuppression, and that allows thymic recovery, is needed.

If the stem cell innoculum is replete, then hematopoietic stem cells are infused with other cells including erythrocytes, donor-tolerized mature lymphocytes, as well as hematopoietic stem cell-derived precursors. The donor-derived T-lymphocytes maybe antigen naïve or experienced and are able to interact with tumor or viral antigen. However, antigen-naïve T-lymphocytes, activated in the post-HSCT millieu, may proliferate and directly attack recipient tissue to cause aGvHD. This initial wave of donor-tolerized T-lymphocyte expansion occurs within the first 120 days. Beyond 120 days, a second wave of T-lymphocyte expansion occurs (or, in the case of a T-lymphocyte depleted donor inoculum, the first wave), as donor stem cell-derived T-lymphocytes that have been tolerized in the recipient thymus are exported into the periphery (4).

Many monogenic disorders of immunity have now been described in patients with primary immunodeficiency (PID). Some of these, including the failure to develop central or peripheral tolerance, as well as the codependence of developing thymocytes and developing thymic stromal cells may give insights into the perpetuation of aGvHD in patients who fail first-line treatment with corticosteroids. This article will review the role of alloreactive T-lymphocytes and treatment of aGvHD in causing thymic damage and apply lessons learnt from the study of patients with PID to patients with resistant aGvHD following allogeneic hematopoietic stem cell transplantation.

## Thymic Structure and Normal Thymopoiesis

The thymus is the primary lymphoid organ responsible for the continuous and life-long production of a functional pool of T-lymphocytes exhibiting a widely diverse TCR repertoire, capable of reacting with harmful foreign antigens, but that also recognizes and tolerates self-antigens. The thymus is divided into the subscapular region, the cortex, the cortico-medullary junction, and the medulla. The major cellular components of the thymic stroma include epithelial cells, dendritic cells (DCs), reticular fibroblasts, and macrophages together forming a specialized three-dimensional microenvironment critical for the recruitment of T-lymphocyte precursors followed by an orderly sequential process of T-lymphocyte development and maturation (5). Thymic epithelial cells (TECs) are the major component of the thymic stromal scaffold, divided into two main compartments—the cortical (c) and medullary (m) TECs that exhibit distinct functional properties. A complete, undisrupted thymic microenvironment is essential for normal T-lymphocyte development (6). Conversely, normal thymic architectural development is dependent on input from the developing thymocytes, so called “thymic crosstalk” (7).

Because the thymus does not contain hematopoietic stem cells, progenitor cells are recruited from the bone marrow and enter the thymus at the cortico-medullary junction, with P-selectin and platelet P-selectin glycoprotein ligand on the progenitor cells appearing to play an important role in this homing process (8). Commitment to the T-lymphocyte lineage occurs following interaction between Notch-1 receptor and delta-like 4 ligand expressed by the cTECs (9). At this stage, thymocytes express a “triple negative” (TN) phenotype, devoid of CD3, CD4, and CD8 surface markers. Following expansion of the TN cells, controlled by signals such as IL-7 and Fms-like tyrosine kinase 3 ligand, they gain both CD4 and CD8 to acquire a “double positive” (DP) phenotype with a heterodimeric TCRαβ complex (10, 11). TCR diversity is generated by random genetic rearrangements of the TCR loci and is estimated to be in the region of 10^20^ α–β chain combinations (5). Because these genetic combinations have the potential to generate self-reactive TCRs, which carry the risk of autoimmunity, thymocytes are subjected to a rigorous two-stage selection process to identify and remove these potentially damaging self-reactive T-lymphocytes (12). The first stage (positive selection) takes place in the cortex where DP thymocytes are exposed to a self-peptide/major histocompatibility complex (MHC) complex presented by cTECs. Thymocytes that recognize this complex with intermediate affinity proceed to the next stage of development, ensuring recognition of antigen in association with self-MHC molecules. If the TCR does not recognize the complex or recognizes with high affinity, the T-lymphocyte will undergo apoptosis or “death by neglect.”

Following positive selection, the surviving thymocytes migrate to the medulla, predominantly regulated by chemokine receptor 7 and the corresponding ligands CCL19 and CCL21 expressed by mTECs, and subsequently lose either CD4 or CD8, dependent on which MHC class they associated with during positive selection, to become “single positive” (SP) cells. The second stage of TCR selection, designated negative selection, occurs in the medulla where SP thymocytes are exposed to a self-peptide/MHC complex presented by mTECs and DCs. Medullary TECs possess the unique ability of ectopic expression of a wide range of peripheral tissue-restricted self-antigens (TRAs). This so called “promiscuous gene expression” is partly controlled by the autoimmune regulator (AIRE) transcription factor, as well as the more recently described Fezf2 transcription factor (13), to form a “molecular mirror of peripheral self” (14, 15). TCRs that react with high affinity to the TRA/MHC complexes are deleted as these have the potential to elicit autoimmunity. Re-encounter of peptides present on both cTECs and mTECs, so called “shared peptides,” also leads to thymocyte deletion, a mechanism thought to increase peptide/MHC diversity (16). Negative selection is an indispensable part of central tolerance, a key process that renders T-lymphocytes tolerant of self.

## Peripheral Tolerance

**Figure 1:**
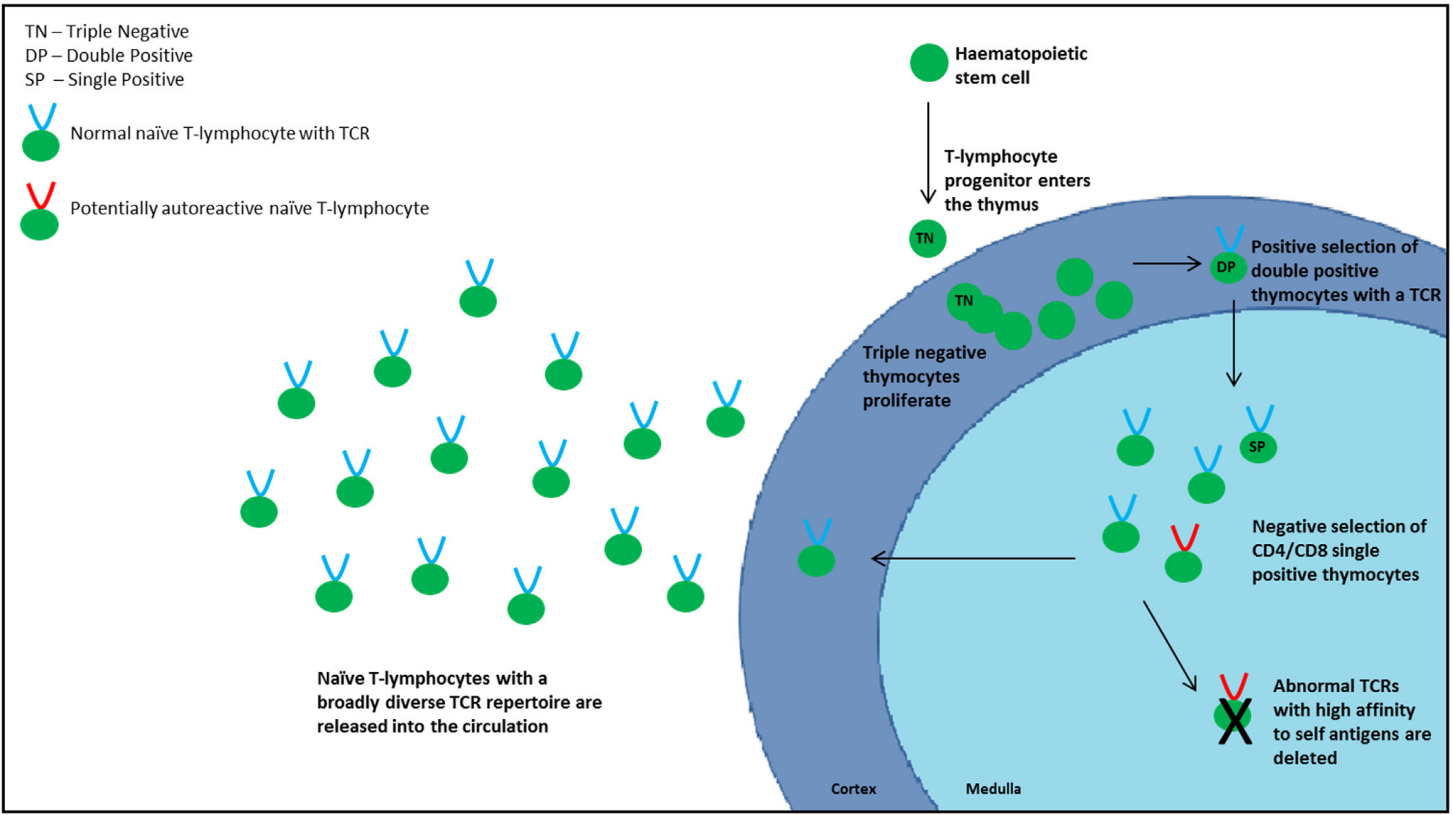
**Normal thymopoiesis**.

The mature surviving thymocytes, termed recent thymic emigrants (RTEs), are exported into the circulation and expand in response to exposure to antigen or homeostatic signals indicating lymphocytopenia (homeostatic peripheral expansion, HPE) (Figure 1). To maintain flexibility in diversity, thymic negative selection is qualified, and some self-reactive T-lymphocytes enter the periphery. Other additional mechanisms are therefore in place to counteract this “escape.” One of these is the production of regulatory T-lymphocytes (Tregs) of which there are two types: natural and inducible. Natural Tregs (nTregs) are produced in the thymus, whereas inducible Tregs (iTregs) are transformed from naïve T-lymphocytes in the periphery upon stimulation. Tregs have an essential role in downregulating peripheral immune responses and limiting inflammation that may be harmful to the host but also in the maintenance of self-tolerance (17). Discrimination between inducible and nTregs is essential to understand fully their specific functions in regulating immune homeostasis as well as their role in different disease states. Expression of Helios has been used as a marker of thymic-derived Tregs, although this has been challenged following the demonstration of induction of Helios expression both *in vitro* and *in vivo* (18). Forkhead box transcription factor P3 (Foxp3) plays a critical role in Treg differentiation. It is not clear exactly how nTregs are generated in the thymus, although autoreactive T-lymphocytes may convert to Tregs rather than undergo apoptosis. The exact mechanisms by which they exert their regulatory effects are also not certain. Proposed mechanisms of Tregs include suppression of T-lymphocyte proliferation, alteration of cytokine production and of CD8^+^, DC, B-lymphocyte, and NK activity. IL-10 and TGFβ are considered to be the central inhibitory cytokines involved in the mechanism of Treg-mediated immunosuppression, but also play a role in the generation of iTregs (19).

## Markers of Thymic Output

The peripheral naïve T-lymphocyte pool is maintained by a combination of proliferation of the circulating naïve CD4^+^ T-lymphocytes and export of naïve T-lymphocytes from the thymus, balanced with apoptosis or differentiation into effector or memory T-lymphocytes. The size of the T-lymphocyte pool is predominantly determined by HPE, but the production of new T-lymphocytes is essential to maintain a broad TCR repertoire. Measuring thymic output provides an indicator of functional T-lymphocyte immunity, and post-HSCT, it indicates reconstitution of the T-lymphocyte compartment.

T-cell receptor excision circles (TRECs) are circular pieces of DNA produced as a consequence of TCRα and TCRβ chain formation. TCRβ chain formation occurs at the TN stage prior to proliferation and generates Dβ-JβTRECs. The TCRα chain is formed at the DP stage of development and generates sjTRECs. Because sjTRECs do not replicate, sjTREC levels become progressively more dilute with naïve T-lymphocyte proliferation. In the setting of post-HSCT lymphocytopenia, this may potentially result in underestimation of the true sjTREC value and thymic activity. However, using PCR, quantification of sjTREC content in T-lymphocytes provides a practical and accepted measurement of thymic output by calculation of the frequency of sjTRECs in a defined population of mononuclear cells or sorted CD4^+^ or CD8^+^ T-lymphocytes (20). A more accurate quantification of thymic output is the sjTREC to Dβ-JβTREC ratio (thymic ratio). This represents intrathymic proliferation that occurs between the TN and DP stages, the main determinant of thymic cellularity, which in turn provides a more accurate estimation of thymic output (21). The thymic ratio has the advantage of not being affected by peripheral T-lymphocyte expansion but use is limited by the fact that it is labor intensive and expensive.

No specific surface markers for RTEs have been identified in humans to date. Naïve T-lymphocyte markers such as CD45RA and CD62L are not always reliable as expansion can occur without loss of these markers (22, 23). In addition, CD45RO^+^ cells can revert back to a CD45RA phenotype. With advances in immunophenotyping techniques, the expression of CD31 (platelet endothelial cell adhesion molecule-1) on naïve CD45RA^+^CD4^+^ T-lymphocytes has been used as a marker for RTEs. CD31^+^CD4^+^ T-lymphocytes were found to have a high sjTREC content and numbers declined with age (21, 24). However, despite RTEs containing a high content of CD31^+^CD4^+^ T-lymphocytes, CD31^+^CD4^+^ T-lymphocytes are not exclusive RTE markers as CD31 is not always lost when naïve T-lymphocyte proliferate and CD31 can also be expressed by other cells including endothelial cells, mast cells, and NK cells (25).

The quality of the T-lymphocyte compartment is best assessed by measuring TCR diversity and T-lymphocyte function. As TCR repertoire diversity is almost completely reflective of the naïve T-lymphocyte compartment, measurement can provide information regarding thymic output (26). A more diverse TCR repertoire is also associated with increased TREC concentrations (3). T-lymphocyte functional tests involve measuring levels of cytokines following T-lymphocyte stimulation or detecting the presence of antigen-specific T-lymphocytes.

## Primary Immunodeficiencies that Impair Central or Peripheral Tolerance

The significance of the mechanisms described above in developing and maintaining efficient, self-limited host defense while preserving self-tolerance is confirmed by studying patients with primary immunodeficiencies. The importance of thymocyte–TEC cross talk is demonstrated in patients with so called “leaky” severe combined immunodeficiency (SCID) with hypomorphic genetic defects affecting hematopoietic stem cells which almost completely block development of early T-lymphocyte precursors. While null mutations completely abrogate T-lymphocyte development, hypomorphic mutations permit a few T-lymphocyte clones to develop. However, patients experience severe T-lymphocytopenia, infection susceptibility, and autoimmunity. Histological examination of the thymus of patients with null and hypomorphic SCID mutations demonstrates severe thymic atrophy, with loss of cortico-medullary demarcation. Additionally, there is severe impairment of TEC progenitors to differentiate into cTEC and mTEC, leading to absence, or reduction of AIRE expression and of FOXP3-expressing Tregs (27). Thymic DCs are absent. These features lead to failure of thymic TRA expression and presentation, failure of positive and negative T-lymphocyte selection, and failure of thymic nTreg development, leading to a dysregulated TCR repertoire, thymic egress of autoreactive T-lymphocytes, and autoimmunity.

Patients with complete DiGeorge syndrome have genetically normal hematopoietic stem cells, but failure of the embryonic third pharyngeal pouch to form the thymus anlage leads to athymia (or in partial DiGeorge syndrome, atopic microthymus). Neural crest-derived mesenchymal cells of the embryonic pharyngeal arches generate the thymic connective tissue. Thymic mesenchyme promotes thymic epithelium development and signaling between mesenchyme and epithelium controls initial thymic morphogenesis. Mesenchymal cells regulate proliferation and differentiation of immature TEC. However, as thymic development becomes able to support immature thymocytes, further thymic epithelial differentiation is essentially independent of mesenchymal cells. Patients with complete DiGeorge syndrome have no T-lymphocytes, because of athymia rather than an intrinsic hematopoietic stem cell defect. Serial transplantation of allogeneic thymus tissue demonstrates subsequent development of normal thymic architecture from thymic epithelial progenitors, as thymocyte progenitors populate the substrate (28). Thymic development is incomplete however, and autoimmunity may be a feature in transplanted patients (29, 30).

Mutations in *AIRE* impair thymic medullary TRA expression, leading to impaired negative selection and subsequent multi-organ autoimmunity. In humans, this manifests as the rare condition autoimmune polyendocrinopathy candidiasis ectodermal dystrophy (15, 31). Similarly, loss of Fezf2 in mouse models leads to autoantibody production and autoimmune disease (13).

Mutations in *FOXP3* are the cause of immune dysregulation, polyendocrinopathy, enteropathy, X-linked syndrome (32). Patients present, usually in early infancy, with autoimmune cytopenias, severe autoimmune enteritis, and type 1 diabetes mellitus. Affected tissues show a T-lymphocyte infiltrate and patients lack functional regulatory FOXP3^+^ T-lymphocytes, causing failure of peripheral tolerance (33). A number of other PIDs in which autoimmunity is a significant feature are associated with a reduction in Tregs, including Omenn syndrome (34), DiGeorge syndrome (35), and CTLA-4 deficiency (36, 37).

## HSCT and T-Lymphocyte Reconstitution

Chemotherapy and/or radiotherapy conditioning is usually given pre-HSCT to remove malignant cells, prevent graft rejection, and make space in the bone marrow for the incoming graft. Subsequently, there is an “aplastic phase,” with obliteration of innate and adaptive immune responses, subjecting the patient to a period of increased risk of infection until donor stem cells engraft and reconstitution of the immune system ensues (38). Recovery of innate immunity occurs relatively quickly, but reconstitution of the adaptive T- and B-lymphocyte compartment is a more lengthy and complex process (2, 39). Incomplete or delayed IR, particularly of the T-lymphocyte compartment, is associated with increased post-transplant morbidity and mortality (40). Thymic function, and consequently thymic output, is negatively affected by advancing age, cytotoxic conditioning pre-HSCT, and GvHD (41). Potential strategies to boost thymic function and promote faster and complete IR, particularly in older patients who exhibit reduced thymic function inherently due to aging, have garnered much interest to improve patient outcome (42).

Restoration of the T-lymphocyte compartment post-HSCT occurs by two parallel pathways (43). Initially after HSCT, the rise in T-lymphocyte numbers is thymic-independent, with expansion of pre-existing surviving host T-lymphocytes or mature donor T-lymphocytes transferred with the graft. However, TCR diversity is dependent upon the repertoire of the initial T-lymphocyte population and expansion results in skewing of the TCR repertoire with time, as well as gradual depletion of T-lymphocytes. This expansion provides an initial degree of immune protection in the post-transplant period, particularly from the host and donor memory T-lymphocytes against re-infection with specific pathogens such as CMV and EBV (26, 44), but is limited in its diversity and permanency, with prevailing susceptibility to infections (41, 45).

Complete and long-lasting IR following lympho-depletion requires durable *de novo* regeneration of naïve T-lymphocytes from donor progenitor cells within the thymus, which exhibit a broad TCR repertoire capable of recognizing a wide range of pathogens (the thymic-dependent pathway) (46, 47). This process is dependent on a functioning and structurally intact thymus to export a regular stream of recipient-tolerized donor stem cell-derived naïve T-lymphocytes. Swift rebuilding of a competent normo-cellular T-lymphocyte compartment is an essential prerequisite for a normal life enabling regular development and function.

## Graft-versus-Host Disease

Despite advances made in the management of HSCT, GvHD remains a leading cause of morbidity and mortality (48), and limits the success and more widespread application of this therapy. The incidence of grade II–IV aGvHD in children ranges from 28 to 56% (49), depending on the degree of histocompatibility, recipient age, underlying condition, and conditioning regimen used (50). Higher aGvHD grades have consistently been associated with worse transplant-related mortality (TRM) and lower overall survival rates (51).

Acute graft-versus-host disease is mediated by donor-tolerized mature T-lymphocytes that recognize and attack disparate host antigens resulting in a harmful inflammatory response. The most important targets are the HLA, encoded by the MHC located on the short arm of chromosome 6, which play a key role in tissue histocompatability and T-lymphocyte recognition (52). The degree of MHC mismatch between donor and recipient is the most important determinant of GvHD, most importantly at the HLA-A, -B, -C, and DRB1 loci (48). However, even in the setting of a HLA-identical sibling HSCT, an alloreactive response can still occur due to mismatch between minor histocompatibility antigens (53).

## Pathophysiology of aGvHD

The Billingham criteria identified three requirements necessary for the development of aGvHD (54):

- The graft must contain immunocompetent cells.
- There must be a disparity between host antigens and those in the graft.
- The host must be unable to launch an immune response against this process.

Elucidation of aGvHD pathophysiology is based on experimental models (55): damage to host tissue by conditioning regimens, underlying disease, and/or infections leads to release of pro-inflammatory cytokines such as IFNγ, TNFα, and IL-1 resulting in an inflammatory environment leading to the activation and maturation of host APCs, and upregulation of adhesion and costimulatory molecules. This cultivates an environment that promotes the recruitment of donor alloreactive T-lymphocytes. Donor T-lymphocytes recognize disparate allo-antigens on activated host APCs and become activated, proliferate, differentiate, produce further inflammatory cytokines, and migrate to target organs directed by chemokines, selectins, and integrins. Effector cells, primarily cytotoxic T-lymphocytes and NK cells, and soluble effectors cause apoptosis of target cells mediated by perforin/granzyme and Fas/Fas ligand pathways (56).

## Clinical Features of aGvHD

Historically, aGvHD was defined as occurring within the first 100 days following HSCT, and chronic (c) GvHD as after 100 days. However, with the development of new strategies such as reduced intensive conditioning, this definition is less clear and a more recent reclassification now includes both late aGvHD occurring after 100 days and overlap syndrome with features of both (Table 1) (57).

**Table 1:** **Classification of GvHD (57)**.

Type		Definition
**Acute**	Classic acute graft-versus-host disease (aGvHD)	Onset ≤100 days post-hematopoietic stem cell transplant (HSCT)/DLI, features of aGvHD
	Persistent/recurrent/late-onset aGvHD	Onset >100 days post-HSCT/DLI, features of aGvHD

**Chronic**	Classic chronic GvHD	Onset at any time post-HSCT/DLI, features of chronic GvHD
	Overlap syndrome	Onset at any time post-HSCT/DLI, features of both acute and chronic GvHD

Acute graft-versus-host disease principally involves the skin, GIT, and liver, with skin manifestations occurring most commonly and usually the earliest following engraftment (48, 55, 58). Patients typically develop a pruritic maculo-papular rash, initially around the neck and shoulders, often involving the palms and soles but sparing the scalp. In severe cases, blistering and ulceration can occur. Gastrointestinal aGvHD usually involves diarrhea but may also manifest as vomiting, nausea, anorexia, abdominal pain, and bleeding. Liver involvement typically manifests as cholestasis due to damage to the bile canaliculi, with elevated alkaline phosphatase and serum bilirubin (Table 2) (59). aGvHD is staged according to the extent of involvement of the skin, GIT, and liver (Table 3) (59). Severe GvHD is associated with a poor prognosis with a 5% long-term survival for grade 4 and 25% for grade 3.

**Table 2:** **Acute graft-versus-host disease staging of individual organ involvement (59)**.

	Stage 0	Stage 1	Stage 2	Stage 3	Stage 4
**Skin**	No rash	Rash <25% of BSA	25–50% BSA	>50% generalized erythroderma	Plus desquamation and bullae

**Gut**	Diarrhea < 10 ml/kg/day	10–19.9 ml/kg/day	20–30 ml/kg/day	>30 ml/kg/day	Severe abdominal pain ± ileus, frank blood, or melena

**UGI**	–	Severe nausea/vomiting	–	–	–

**Liver**	Bilirubin ≤2 mg/dL	2.1–3 mg/dL	3.1–6 mg/dL	6.1–15 mg/dL	>15 mg/dL

**Table 3:** **Overall acute graft-versus-host disease (aGvHD) grading: modified Glucksberg grade (59)**.

Overall aGvHD grade	Skin stage	Liver stage	GIT stage	Upper GI stage
Grade I	1–2	0	0	0
Grade II	3	1	1	1
Grade III	–	2–3	2–4	–
Grade IV	4	4	–	–

## Effect of aGvHD on Thymic Structure, Function, and the T-Lymphocyte Compartment

Although aGvHD principally involves the skin, GIT, and liver, the thymus is also a primary target, resulting in disruption of the thymic architecture. Thymic aGvHD has been shown to cause loss of demarcation between the cortico-medullary zones, loss of Hassall’s corpuscles, alteration of TEC subpopulations, and depletion of thymocytes (60–62). The structural damage to the thymic microenvironment consequently impairs lymphocyte formation and export, reflected by lower TREC levels and a distorted TCR repertoire observed in patients, and occurs independent of age (20, 61, 63–65). aGvHD also has detrimental effects on the thymic-independent pathway with reduced expansion of transferred mature donor-tolerized T-lymphocytes possibly due to loss of peripheral T-lymphocyte niches (65). The thymus appears to be particularly sensitive to the effects of GvHD with thymic output being significantly affected even in grade 1 disease (26). Subclinical thymic GvHD may even occur in the absence of overt aGvHD (66) with an underappreciated adverse effect on reconstitution of adaptive immunity, causing ongoing infections and incomplete IR post-HSCT.

Although the precise mechanisms behind how aGvHD causes thymic damage in humans remain incompletely understood, experimental aGvHD models have helped to delineate the cellular and molecular mechanisms underlying thymic injury and effects on T-lymphocyte development (60). TECs act as initiators and targets of thymic aGvHD, capable of directly activating alloreactive donor T-lymphocytes independently of APCs (63). Activation of alloreactive donor T-lymphocytes causes IFNγ secretion and stimulation of a STAT1-induced apoptosis pathway resulting in death of TECs (63). The resulting disruption of normal thymic architecture and organization of the microenvironment interrupts the normal signals required for immature thymocytes, leading to thymic atrophy and reduced thymic export. Murine models show that thymocyte damage occurs at two stages of development primarily resulting in loss of DP thymocytes. The first stage involves failure of normal TN thymocyte proliferation, thus failing to produce sufficient numbers of DP thymocytes (60, 61). The second stage is increased apoptosis of DP thymocytes (61, 67). Both events contribute to the reduction in thymic lymphoid cellularity, consequent thymic atrophy, and reduced thymic export. Patients with aGvHD show a decrease in βTREC and sjTREC levels, suggestive of an interference at an early developmental stage (pre-TN thymocyte proliferation) either involving early thymocyte precursors or bone marrow-derived progenitors (20, 21).

**Figure 2:**
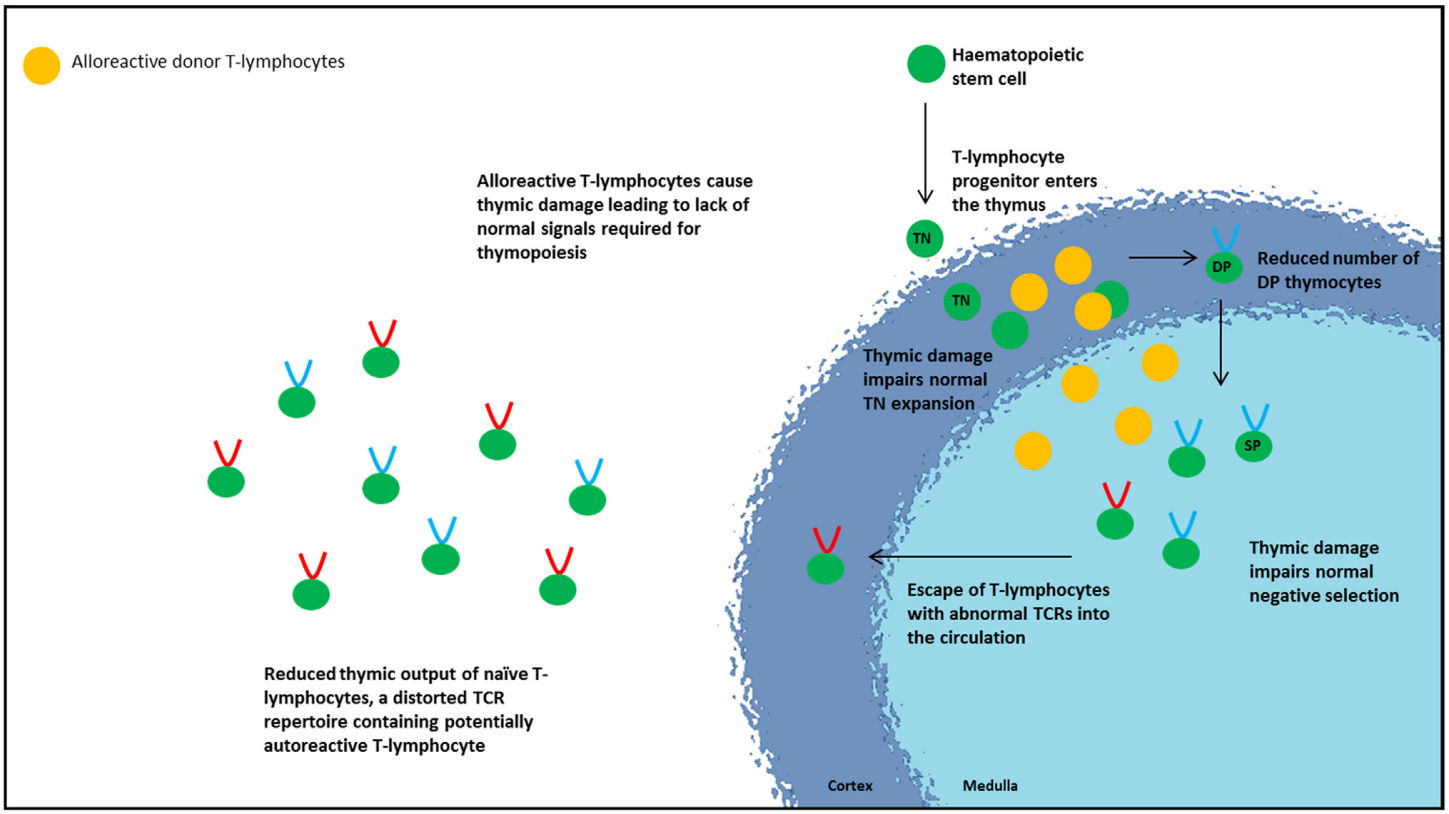
**Effect of acute graft-versus-host disease and corticosteroids on thymic function**.

A distorted TCR repertoire is observed in patients with aGvHD (20). Normally, all thymic stromal cells exhibit the same MHC haplotype. Following HSCT with HLA mismatch, the radio/chemoresistant cTECs continue to express recipient MHC while recipient medullary DCs will be replaced by donor medullary DCs expressing donor MHC molecules. This MHC disparity disturbs thymic positive and negative selection impacting on TCR selection, resulting in thymocytes escaping negative selection, increasing the survival of reactive T-lymphocytes (68–71). It is interesting to speculate whether donor stem cell-derived T-lymphocytes, inadequately tolerized in the recipient thymus are “allo-reactive” or “auto-reactive.” Murine models demonstrate that damage to mTECs by donor CD8^+^ T-lymphocytes disrupts normal thymic negative selection with escape of autoreactive CD4^+^ T-lymphocytes into the circulation (72). Thus, aGvHD is detrimental to quantity and quality of T-lymphocyte recovery. Thymic injury from aGvHD resulting in disruption of the normal negative selection process, and thymic Treg development alters the TCR repertoire and promotes escape of autoreactive cells into the circulation, setting the scene for autoimmunity as seen in cGvHD (72) (Figure 2). It is well established that aGvHD predisposes to cGvHD but the mechanistic link between them has been uncertain. Recently, Dertschnig et al. demonstrated that impaired thymic ectopic TRA expression secondary to damaged AIRE-expressing mTECs results in disruption to negative selection permitting *de novo* production of TRA-specific T-lymphocytes which escape into the periphery. TRAs most affected were those that are expressed in tissues known to be targets in cGvHD thus providing a potential link between allo-immunity to the development of autoimmunity (73, 74).

## Corticosteroids and Thymic Function

First-line treatment of aGvHD is corticosteroids, which exhibit potent immunosuppressive and anti-inflammatory effects. Although they are effective anti-inflammatory agents, they have significant unwanted effects, including increased risk of cardiovascular disease, osteoporosis, and insulin resistance (75–77). Despite their successful application in some patients, a complete response is only witnessed in 25–50% of patients with aGvHD. Short intensive corticosteroid courses in avian models induce thymic involution and cause a profound reduction in naïve T-lymphocyte production, although with complete recovery following cessation of corticosteroid treatment (78). However, the effects of long-term corticosteroid use in human thymus are unknown. Both aGvHD and immunosuppressive treatment of aGvHD concurrently impair thymopoiesis subjecting the patient to increased risk of infection and other complications.

Patients who are refractory to corticosteroid treatment have an unfavorable prognosis with increased TRM (79). While corticosteroid are well established as first-line therapy for aGvHD, there is no established consensus to standard second-line therapy for patients with steroid-refractory disease or steroid-dependency and usually involves intensification of systemic immunosuppression with a broad plethora of different therapeutic agents such as mycophenolate mofetil, anti-TNFα antibodies, or mammalian target of rapamycin inhibitors (80). These agents mainly non-selectively target T-lymphocytes resulting in a general immunosuppressive effect, and also likely negatively affect the graft-versus-tumor effect (49).

The insights gained from studying the effects of abnormal T-lymphocyte and thymic development in patients with primary immunodeficiencies are instructive in considering effective treatment of aGvHD. To restore normal immunity, early neutralization of the donor-tolerized T-lymphocytes is required to interrupt target-organ damage, particularly to the thymus, while restoring normal thymic architecture which will facilitate appropriate tolerization of donor stem cell-derived thymocytes to the recipient, with adequate elimination of cells likely to cause autoimmunity. In view of the effect of corticosteroids on thymic architecture and function, the rapid cessation of corticosteroid therapy while maintaining control of alloreactive T-lymphocytes would be desirable.

## Extracorporeal Photopheresis (ECP)

Extracorporeal photopheresis involves the collection of peripheral blood mononuclear cells by apheresis, exposure to the photoactive drug 8-methoxypsoralen (8-MOP), and UVA radiation, followed by re-infusion of the photo-activated cells back into the patient (81). The clinical efficacy, safety, and tolerability of ECP in the treatment of aGvHD in patients have been demonstrated in several studies, which also show the steroid-sparing effect (82–85). A systematic analysis of prospective studies examining ECP treatment outcomes in corticosteroids refractory/dependent/intolerant aGvHD in adults and children found an overall response rate of almost 70% in all organs, an encouraging result compared to other second-line treatments used (86). Based on the evidence available, the UK Expert Photopheresis Group guidelines state that ECP should be considered as second-line therapy for patients with aGvHD grades II–IV who are steroid refractory/dependant/intolerant. ECP is given as one cycle weekly (two consecutive days) and is recommended for a minimum of 8 weeks (87). Adverse effects of ECP treatment are minimal and predominantly related to central venous access. The mechanistic actions of ECP have not been fully elucidated but likely immune-modulate adaptive and innate immunity, predominantly acting through DCs and Tregs (88, 89).

Exposure to 8-MOP/UVA results in the formation of covalent bonds with pyrimidine bases and subsequent cross-linking of DNA, inducing apoptosis of the exposed cells, with activated T-lymphocytes preferentially affected (81, 90, 91). Apoptosis occurs several hours after ECP and peaks on day 3 (92), possibly due to increased Fas-mediated pro-apoptotic signaling (93). However, as only 5–10% of lymphocytes are exposed during the procedure, an insufficient number to entirely account for the effects of ECP, and considering also that the majority of activated T-lymphocytes reside in the tissues rather than the blood, it is speculated that the ECP-exposed cells have indirect immune-modulatory actions on other non-exposed immune-competent cells.

Following ECP, phagocytosis of the apoptotic cell fragments leads to an immune response directed against alloreactive donor T-lymphocytes.

Monocytes undergo apoptosis more slowly than lymphocytes following ECP (94). ECP promotes differentiation of exposed monocytes to DCs (95), stimulated by the physiological interaction of monocytes with adherent platelets during passage through the ECP chamber (95, 96). Although by day 6 post-ECP 80% of monocytes are apoptotic, functional abilities such as T-lymphocyte stimulation, differentiation into DCs, and endocytosis are preserved, despite impairment of migratory capacities (97). As the majority of DCs typically reside in the tissues, this differentiation of monocytes introduces a much larger number of DCs into the circulation than is normally seen, thus increasing the antigen-presenting capacity. Following ECP, apoptotic cells are localized primarily in the liver and spleen, regions rich with DCs, which ingest the apoptotic alloreactive peptide fragments (98). In aGvHD, phagocytosis of ECP-exposed apoptotic cells results in DCs acquiring an immature tolerogenic state, characterized by downregulation of maturation markers and costimulatory molecules such CD40, CD80, CD83, and CD86 and increased secretion of anti-inflammatory cytokines such as TGFβ and IL-10, resulting in enhanced phagocytic activity but a reduced ability to stimulate an effector T-lymphocyte immune response (91, 99–102). IL-10 is a key player in immune downregulation and induction of tolerance, specifically by preventing DC maturation and generating Tregs (91). Monocyte-derived immature DCs also show upregulated expression of the glucocorticoid-induced leucine zipper gene, a marker of tolerogenic DCs, following ECP exposure (103). Upon interaction with T-lymphocytes, tolerogenic DCs can induce anergy or apoptosis, or stimulate the production of Tregs. However, these DCs are not confined to this immature state and can respond to inflammatory signals such as lipopolysaccharide resulting in full maturation (91).

## Tregs and ECP

The generation of Tregs is an important immunomodulatory action of ECP. In aGvHD murine models, ECP-treated splenocytes improved aGvHD and IR by reducing the number of non-exposed CD8^+^ effector lymphocytes, suppressing allogeneic T-lymphocyte proliferation and increasing the number of Tregs (104).

Ten patients with acute and chronic GvHD showed a significant increase in Tregs following ECP, which was accompanied by increased glucocorticoid-induced tumor necrosis factor receptor-related protein expression (105). A larger study involving 27 patients with acute and chronic GvHD showed a significant increase in Treg numbers in those who responded to ECP treatment (106).

## Conclusion

In approaching treatment of aGvHD, tipping the balance toward immune tolerance rather than immune suppression and reducing thymic aGvHD, as well as decreasing the burden of immunosuppressive medications, could conceivably allow regeneration of thymic function, as suggested in preliminary evidence by Beattie et al. (107). In this case report of a single patient with aGvHD, there was a temporal association of the commencement of ECP and reduction in corticosteroid dose with a rise in thymic export and Tregs. Further studies are required to substantiate this observation, but if confirmed, ECP would seem an attractive treatment option for aGvHD, given the lack of global immunosuppression with preservation of adaptive immune responses to novel and recall antigens.

## Author Contributions

AG conceived the review. AF and AG wrote the review.

## Conflict of Interest Statement

The authors declare that the research was conducted in the absence of any commercial or financial relationships that could be construed as a potential conflict of interest.
